# Trends in prevalence of anaemia among people living with HIV in the UK: 20 cross‐sectional analyses using population‐based electronic primary healthcare records

**DOI:** 10.1111/hiv.70044

**Published:** 2025-05-21

**Authors:** George B. Freer, Jennifer Cooper, Krishnarajah Nirantharakumar, G. Neil Thomas, Tiffany E. Gooden

**Affiliations:** ^1^ Department of Applied Health Sciences University of Birmingham Birmingham UK

**Keywords:** anaemia, comorbidities, epidemiology, HIV, trend analysis

## Abstract

**Background:**

People living with HIV have a greater prevalence of anaemia compared with people without HIV, which increases the risk of associated morbidity and premature mortality. Risk factors for anaemia among people living with HIV have changed in recent decades due to new antiretroviral therapy (ART), increased uptake of ART and increasing chronic conditions among people living with HIV; thus, anaemia prevalence may have changed over time. We aimed to identify the prevalence and trends of anaemia among people living with HIV over a 20‐year period.

**Methods:**

A series of 20 annual cross‐sectional analyses were performed from 2002 to 2021. Data on people living with HIV aged ≥18 years from Clinical Practice Research Datalink (CPRD) Aurum was used, a population‐based UK primary healthcare database. Overall and annual prevalence of all‐cause anaemia, defined as any clinical code indicative of having anaemia, was calculated using multivariable logistic regression models and adjusted for age, sex, ethnicity, smoking status and deprivation. Trends were determined by investigating the change in prevalence across the 20 annual analyses using linear regression. Prevalence and trends of anaemia were also calculated among the following sub‐groups of people living with HIV: age, sex, ethnicity, body mass index, smoking and socioeconomic deprivation.

**Results:**

Data for 41 990 people living with HIV were included. Overall adjusted prevalence of anaemia was 6.89%; however, this significantly increased from 4.6% (95% CI: 4.72%, 5.06%) in 2002 to 7.42% (95% CI: 7.33%, 7.51%) in 2021. A significant positive trend for anaemia was found (adjusted coefficient + 0.123; 95% CI: 0.107, 0.139; *p* < 0.001) and this was consistent among all sub‐groups. Females, older age (≥50 years), non‐smokers, Black ethnicity, overweight/obese and higher deprivation had an increased prevalence of anaemia.

**Conclusion:**

Anaemia among people living with HIV is increasing, with certain groups of people living with HIV experiencing a greater burden. Efforts should be made to prevent and reduce anaemia among people living with HIV to mitigate further morbidity, premature mortality and additional inequalities.

## BACKGROUND

The incidence of HIV has decreased in the last two decades; however, prevalence is increasing with 39.9 million people living with HIV globally at the end of 2023 [1]. This is in part due to improved and higher uptake of antiretroviral therapy (ART), resulting in people living with HIV having a life expectancy reaching that of the general population [2]. Despite these successes, an ageing population of people living with HIV has led to the emergence of various age‐related comorbidities [3, 4, 5, 6, 7].

Anaemia is a common condition among people living with HIV, and is associated with increased morbidity and premature mortality [8, 9, 10]. Anaemia is a deficiency in mature red blood cells (RBCs) required to deliver oxygen to respiring tissue around the body [11], defined by the World Health Organization (WHO) as haemoglobin <130 g/L in men and <120 g/L in non‐pregnant women [12]. Several factors have been identified that may influence the prevalence and severity of anaemia among people living with HIV. HIV can lead to a decreased RBC production by bone marrow infiltration or a blunted response to endogenous erythropoietin [13]. Likewise, coinfections, opportunistic infections and certain ARTs have been known to increase the development of anaemia among people living with HIV [13]. A more advanced stage of HIV, indicated by high viral loads and low white cell counts, amplifies these processes; thus, anaemia presents more often and with greater severity among people living with HIV with unsuppressed viral load [14]. Although ART reduces this risk, some ART drugs are associated with increased rates of anaemia, such as zidovudine and stavudine [15, 16]. Similar to the general population, increasing age, female sex and Black ethnicity have also been linked to increased prevalence of anaemia among people living with HIV [13, 14, 17, 18, 19].

ART use among people living with HIV increased from 70% in 2008 [20] to 99% in 2021 in the UK [21]. Consequently, viral load has reduced along with opportunistic infections due to improved immune systems among people living with HIV. However, even when viral load is low, people living with HIV experience chronic inflammation within the bone marrow, which can cause abnormalities in blood cell production [22, 23]. Although the safety and quality of ART has also improved over the last 15 years, with older forms of ART that are associated with anaemia no longer recommended as first‐line therapies (e.g., zidovudine and stavudine) [24], it is unclear what impact the increased duration of living with HIV (and the associated negative direct and indirect biological impacts) and being exposed to ART has had on the development of anaemia. Additionally, the burden of age‐related conditions associated with anaemia (e.g., chronic kidney disease) has increased among people living with HIV, for which they are at higher risk and such conditions occur at an earlier age compared with people without HIV [7, 25]. There is also evidence to suggest that lifestyle‐related risk factors associated with anaemia may have increased among people living with HIV in the UK (e.g., smoking, alcohol and substance use) [26, 27, 28, 29].

Due to the drastic changes among these complex factors and mechanisms over the last two decades, it is unclear how the burden of anaemia among people living with HIV may have been altered. Identifying any trends would enable optimal service planning and resource allocation to prevent, diagnose, and treat anaemia among people living with HIV accordingly. In turn, reduced symptoms of anaemia (e.g., fatigue, shortness of breath), related morbidity and premature mortality, and improved quality of life among people living with HIV could be achieved.

A 2022 systematic review and meta‐analysis [30] reported the prevalence of anaemia in adult people living with HIV from 47 observation studies (10 conducted in North America or Europe), with the prevalence ranging from 21.2% to 79.1%. However, 35 of the 47 studies had small sample sizes (*n* < 1000), and most did not use population‐based recruitment methods [30]. This meta‐analysis reported an overall prevalence of anaemia of 47%; however, the validity of this analysis can be questioned given the considerable heterogeneity in included studies (*I* ^2^ = 99.5%). Only three studies (conducted in 1999, 2008, and 2014) exist that included, but were not exclusive to, people living with HIV in the UK; each of these studies combined data from various prospective cohorts of people living with HIV and reported a prevalence of 60%, 35%, and 28%, respectively [31, 32, 33]. Given these studies were conducted more than 10 years ago and the burden among UK‐based people living with HIV cannot be ascertained from these multi‐country results, the current burden among people living with HIV in the UK is unknown.

To our knowledge, only three studies exist that report trends in anaemia among people living with HIV; two were conducted from the North American AIDS Cohort Collaboration on Research and Design (NA‐ACCORD) cohort data from the United States and Canada, and one was conducted in rural China [34, 35, 36]. Two of these studies found a decreasing trend: from 44% to 28% between 2012 and 2016 in China [36], and from 33% to 20% between 2007 and 2017 in North America [34]. The third study found that people living with HIV and anaemia had a much higher risk of death, and those that died during the study period (2007–2016) experienced a decreasing trend in haemoglobin levels within the 4 years prior to death [35]. These existing studies have limited generalisability; for instance, data from NA‐ACCORD comprises mostly males (88%) [34], and the cohort from China comprised mostly farmers, all of Han ethnicity, and 85% of the sample was diagnosed with HIV following contaminated blood donation [36]. It is crucial to understand local trends to inform national policy and recommendations on practice. Currently, the trends of anaemia among people living with HIV in the UK are unknown.

We aimed to determine the overall prevalence and trends in prevalence of anaemia among people living with HIV in the UK from 2002 to 2021 using a population‐based dataset. Additionally, we aimed to determine the overall prevalence and trends among various sub‐groups of people living with HIV.

## METHOD

### Study design and data source

A series of 20 cross‐sectional analyses were conducted from 1 January 2002 to 31 December 2021. Each cross‐sectional analysis comprised a 12‐month period from 1 January to 31 December for each year within the study period. Study reporting follows the Strengthening the Reporting of Observational Studies in Epidemiology (STROBE) guidelines [37].

Data for this study were sourced from Clinical Practice Research Datalink (CPRD) Aurum [38], a population‐based database of UK primary healthcare records. At the time of data extraction, CPRD contained more than 40 million patient records from 1491 UK general practices (GPs) [39]. Ninety‐five percent of the UK population is registered with a GP [40], as GPs provide a free service and are typically the first point of contact for a referral to secondary healthcare services in the UK under the National Health Service. CPRD Aurum has been found to be representative of the UK general population in terms of sociodemographic and clinical characteristics [38]. Medical diagnoses, tests and observations are organized in CPRD by local Egton Medical Information System (EMIS) codes, Read codes and/or SNOMED‐CT codes. These codes are standardized hierarchical clinical coding systems used in all UK GPs within the National Health Service [41].

Data were extracted from CPRD Aurum in February 2023 using the Data Extraction for Epidemiological Research (DExtER) tool, a validated computerized tool for data extraction [42]. Data were linked with Index of Multiple Deprivation (IMD) [43] data to include a metric of deprivation. IMD is the official UK metric of deprivation [44] and is calculated by matching postcodes with the appropriate quintiles of area‐level socioeconomic status.

### Study population

Adults aged 18 years or older with a code indicative of an HIV diagnosis and who had been registered with a participating GP for at least 12 months were eligible (to ensure accurate data collection). HIV diagnosis was determined through relevant diagnostic codes predetermined by a practicing GP (JC) and checked by another practicing GP; the prevalence of HIV within primary care data is similar to national statistics, and the code list we used has been used in previous studies [7, 25, 45] (Data S1). Participants were excluded if the date of HIV diagnosis occurred after the study end date (31 December 2021), or if they died or transferred to a non‐participating GP before the study start date (1 January 2001). We were unable to identify pregnancies within our dataset; therefore, we did not exclude pregnant women from our analyses.

### Outcomes

The primary outcome was the overall prevalence and trends of anaemia among people living with HIV within the 20‐year study period. Given the overuse of generic codes among GPs, all‐cause anaemia was used to define the anaemia outcome. All‐cause anaemia was used as a binary outcome variable, defined by any code indicative of a diagnosis of anaemia on or before the date of eligibility (i.e., study start date, date of clinical code indicative of living with HIV, date they turned 18 years old or date they had been registered with a GP for at least 12 months). Where a person had more than one clinical code indicative of anaemia, only the one closest to the date of eligibility was used. The full code is provided in Data S1. From discussions with our internal clinical team of GPs, it was decided to use clinical codes to define our outcome instead of haemoglobin, and this is because haemoglobin measurements are not well recorded in primary healthcare records for people living with HIV; however, clinical codes pertaining to diagnosis, conditions and test outcomes are. Previous research indicates that anaemia may be underestimated in CPRD due to investigations only taking place when there is a clinical reason to do so [46]; however, people living with HIV are likely to have more regular health checks in comparison with the general population.

Overall prevalence and trends of anaemia were also assessed among the following sub‐groups of people living with HIV: age (<50, ≥50 years), sex (male, female), ethnicity (White, Black, other), body mass index (BMI) (underweight, normal weight, overweight/obese), smoking (never smoked, current/ex‐smoker) and socioeconomic deprivation (most deprived, least deprived). BMI was coded in accordance with WHO's guidance thresholds of obesity [47] where underweight is defined as a BMI <18.5 kg/m^2^, normal weight is defined as a BMI 18.5–24.9 kg/m^2^ and overweight/obese is defined as a BMI ≥25 kg/m^2^. The most deprived category was defined as IMD quintiles 4 and 5, and the least deprived category was defined as IMD quintiles 1, 2 and 3. Sub‐groups were chosen based on clinical importance and existing literature [13, 18] regarding prognostic factors of anaemia.

### Statistical analysis

All statistical analyses were performed using STATA version 17.0 and R version 4.4.3. For all statistical tests, significance was set at *p* < 0.05. Descriptive statistics were used to present baseline characteristics. Continuous variables were normally distributed and reported with mean and standard deviation (SD). Categorical variables were reported as frequency and percentage.

To estimate the adjusted prevalence of anaemia, we fitted a multivariable logistic regression model. We adjusted for age (continuous), sex (male, female), ethnicity (White, Black, others), smoking status (current/ex‐smoker, never smoked), BMI (underweight, normal weight, overweight/obese) and deprivation (most deprived, least deprived). To maintain the originality of the data, missing data were included as an additional category where necessary (i.e., for ethnicity, smoking status, BMI and deprivation). Adjusted prevalence was calculated for each calendar year from 2002 to 2021 by using the fitted model to calculate the mean predicted probability for individuals meeting the eligibility criteria for each respective year. To generate 95% CIs, a non‐parametric bootstrap procedure was used, sampling with replacement 1000 times. For sub‐group analyses, this procedure was repeated within strata defined by each sub‐group investigation, including their respective missing indicator variables to ensure consistency with the main model. A sensitivity analysis was conducted where we calculated overall prevalence using a complete case analysis to determine whether missing data altered the results.

Each annual cross‐sectional analysis included individuals with a diagnosis of HIV occurring during or before the year of analysis. People living with HIV were excluded if death or transfer to a non‐participating GP occurred before the start date of each annual analysis. For example, the 2007 analysis included anyone with a diagnostic code indicative of an HIV diagnosis on or before 31 December 2007, and anyone who died or transferred to a non‐participating GP before 1 January 2007 was excluded; this formed the denominator for calculating the 2007‐point prevalence. Presence of anaemia was considered if the diagnosis occurred before the end date of each annual analysis. For example, the 2007 analysis included any people living with HIV with an anaemia diagnosis made on or prior to 31 December 2007; this formed the numerator for calculating the 2007‐point prevalence.

Trends were modelled over time as a linear regression and presented with regression coefficient and 95% CIs. Data were linear in nature and Breusch–Godfrey test [48] identified no autocorrelation, *p* > 0.05. The SD of residuals was plotted on a histogram, which identified no issues with heteroscedasticity.

## RESULTS

### Sample characteristics

**FIGURE 1:**
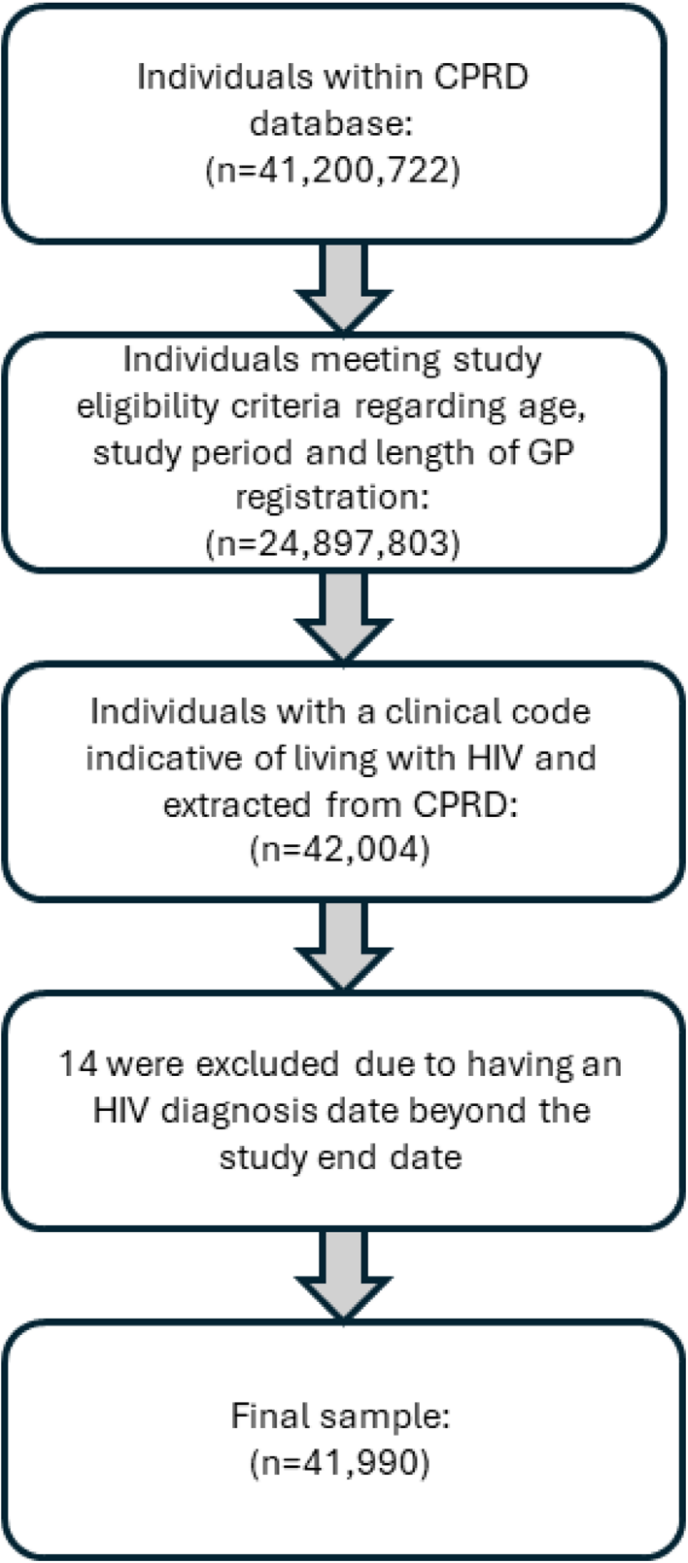
Flow chart of study participants.

All GPs contributing to CPRD Aurum (*n* = 1491) were included in the data extraction. In total, 41 200 722 patient records were available, of which 24 897 803 were eligible for inclusion based on the study period, population age and data quality requirements (Figure 1). From these, 42 004 individuals had a diagnostic code for HIV; however, 14 were excluded due to having an HIV diagnosis date beyond the study end date, leaving a total sample of 41 990 people living with HIV included in this study.

The sample contained a larger proportion of males than females (68% vs. 32%, respectively) and was mostly aged <50 years (81%) (Table 1). People from White ethnic groups represented 44% of the sample, and people from Black ethnic groups made up the next largest proportion (29%); however, ethnicity data was missing for 17% of the sample. A similar proportion of people living with HIV were missing data for BMI (21%); 39% had a normal BMI and 37% had an overweight/obese BMI. Nearly half had never smoked (46%), but this information was missing for 26% of the sample. The sample comprised mostly people living with HIV within the most deprived quintiles (62%).

**TABLE 1:** Baseline characteristics.

	All PLWH (2002–2021) *N* = 41 990	PLWH 2002 *N* = 3217	PLWH 2021 *N* = 19 808	Difference between 2002 and 2021
Sex, *N* (%)				
Male	28 435 (68)	2374 (74)	13 537 (68)	−5.5
Female	13 553 (32)	843 (26)	6269 (32)	5.5
Missing	2 (0)	0 (0)	2 (0)	
Age (mean, SD)	40.7 (10.9)	39.9 (9.8)	42.4 (11.1)	2.5
Age group, *N* (%)				
<50 years	34 049 (81)	2796 (87)	15 020 (76)	−11.1
≥50 years	7941 (19)	421 (13)	4788 (24)	11.1
Missing	9 (0)	0 (0)	0 (0)	
Ethnicity, *N* (%)				
White	18 598 (44)	1040 (32)	9679 (49)	16.6
Black	12 146 (29)	445 (14)	6188 (31)	17.4
Others	4177 (10)	120 (4)	2403 (12)	8.4
Missing	7069 (17)	1612 (50)	1538 (8)	−42.3
BMI category, *N* (%)				
Underweight	1167 (3)	89 (3)	521 (3)	−0.1
Normal	16 530 (39)	1176 (37)	7569 (38)	1.6
Overweight/Obese	15 561 (37)	617 (19)	8143 (41)	21.9
Missing	8732 (21)	1335 (41)	3575 (18)	−23.4
Smoking status, *N* (%)				
Current/ex‐smoker	11 878 (28)	595 (19)	9644 (49)	30.2
Never smoked	19 282 (46)	359 (11)	6352 (32)	20.9
Missing	10 830 (26)	2263 (70)	3812 (19)	−50.8
IMD, *N* (%)				
Least deprived	14 447 (34)	1094 (34)	6995 (35)	1.3
Most deprived	25 852 (62)	1942 (60)	12 243 (62)	1.4
Missing	1691 (4)	181 (6)	570 (3)	−2.8

Abbreviations: BMI, body mass index; IMD, index of multiple deprivation; PLWH, people living with HIV; SD, standard deviation.

Several changes in demographics across the study period are worth noting (Table 1). From 2002 to 2021, the proportion of females increased from 26% to 32%. The average age increased by 2.5 years from 39.9 to 42.4 years, with the proportion of people living with HIV aged ≥50 years nearly doubling from 13% to 24%. There was a large increase in the proportion of people living with HIV who were classified as overweight/obese by BMI from 19% to 41%, likely reflective of national trends. There was a large decrease in the proportion of missing data for ethnicity (50%–8%) and smoking status (70%–19%) between 2002 and 2021, resulting in an increase of White and Black people living with HIV, and an increase in both current/ex‐smokers and those who had never smoked.

### Overall prevalence of anaemia

The overall adjusted prevalence of anaemia (over the 20‐year study period) was 6.9%, with 2894 people living with HIV having a clinical code indicative of anaemia (Table 2); this increased to 7.94% (95% CI 7.87%–8.02%) in a complete case analysis. Males living with HIV demonstrated a significantly lower prevalence of anaemia compared with females living with HIV (3.84% vs. 13.30%, *p* < 0.001). People living with HIV with a diagnosis of anaemia were on average 4 years older than those without anaemia (44.3 vs. 40.4 years, *p* < 0.001). People living with HIV aged <50 years demonstrated a significantly lower prevalence of anaemia compared with people living with HIV aged ≥50 years (5.99% vs. 10.75%, *p* < 0.001). People living with HIV from Black ethnic groups had a significantly (*p* < 0.001) greater prevalence of anaemia (11.12%) compared with White people living with HIV (4.58%) and other (7.97%). For BMI, people living with HIV considered normal weight had a lower prevalence compared with those underweight and overweight/obese (6.00%, 9.17%, 8.73%, respectively, *p* < 0.001). People living with HIV who have never smoked showed a significantly (*p* < 0.001) greater prevalence of anaemia compared with current/ex‐smokers (9.01% vs. 6.24%, respectively). The prevalence of anaemia increased with deprivation, with the least deprived people living with HIV having a prevalence of 5.53% and the most deprived people living with HIV having a prevalence of 7.96%.

**TABLE 2:** Prevalence of anaemia among PLWH, 2002–2021.

	Total PLWH (*N*)	PLWH with anaemia (*N*)	Prevalence of anaemia (%)	*p*‐value
Total sample	41 990	2894	6.89	—
Sex				
Male	28 435	1091	3.84	<0.001
Female	13 553	1803	13.30	
Missing	2	0	0.00	
Age group				
<50 years	34 049	2040	5.99	<0.001
≥50 years	7941	854	10.75	
Missing	0	0	0.00	
Ethnicity				
White	18 598	851	4.58	<0.001
Black	12 146	1351	11.12	
Others	4177	333	7.97	
Missing	7069	359	5.08	
BMI category				
Underweight	1167	107	9.17	<0.001
Normal	16 530	992	6.00	
Overweight/Obese	15 561	1358	8.73	
Missing	8732	437	5.00	
Smoking status				
Current/ex‐smoker	11 878	741	6.24	<0.001
Never smoked	19 282	1738	9.01	
Missing	10 830	415	3.83	
IMD				
Q1 (least deprived)	2895	160	5.53	<0.001
Q2	4223	244	5.78	
Q3	7329	400	5.46	
Q4	13 259	820	6.18	
Q5 (most deprived)	12 593	1002	7.96	
Missing	1691	268	15.85	

Abbreviations: BMI, body mass index; IMD, index of multiple deprivation; PLWH, people living with HIV.

### Trends in the prevalence of anaemia

**FIGURE 2:**
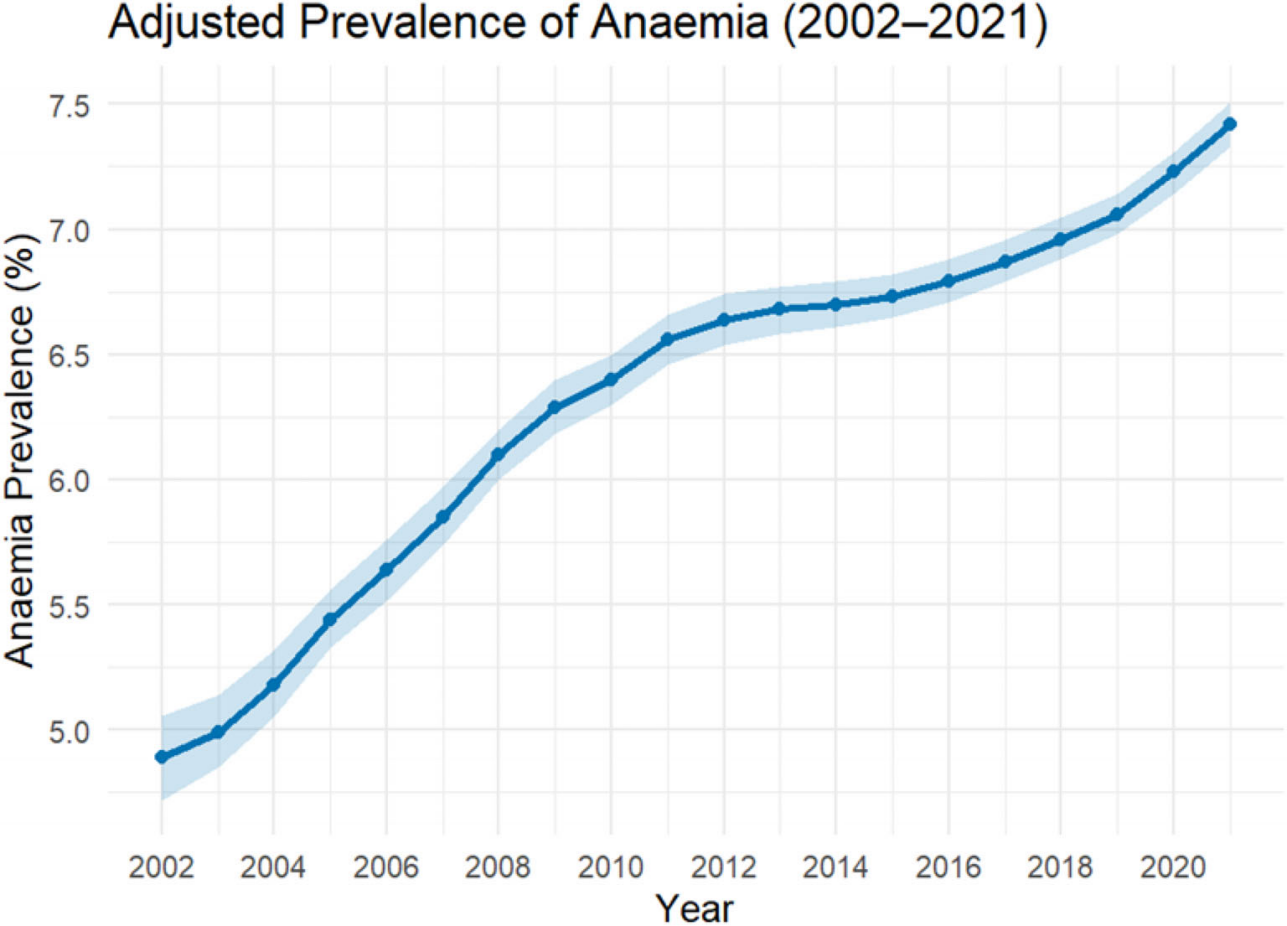
Prevalence of anaemia in PLWH between 2002 and 2021, with 95% confidence intervals and regression line.

**FIGURE 3:**
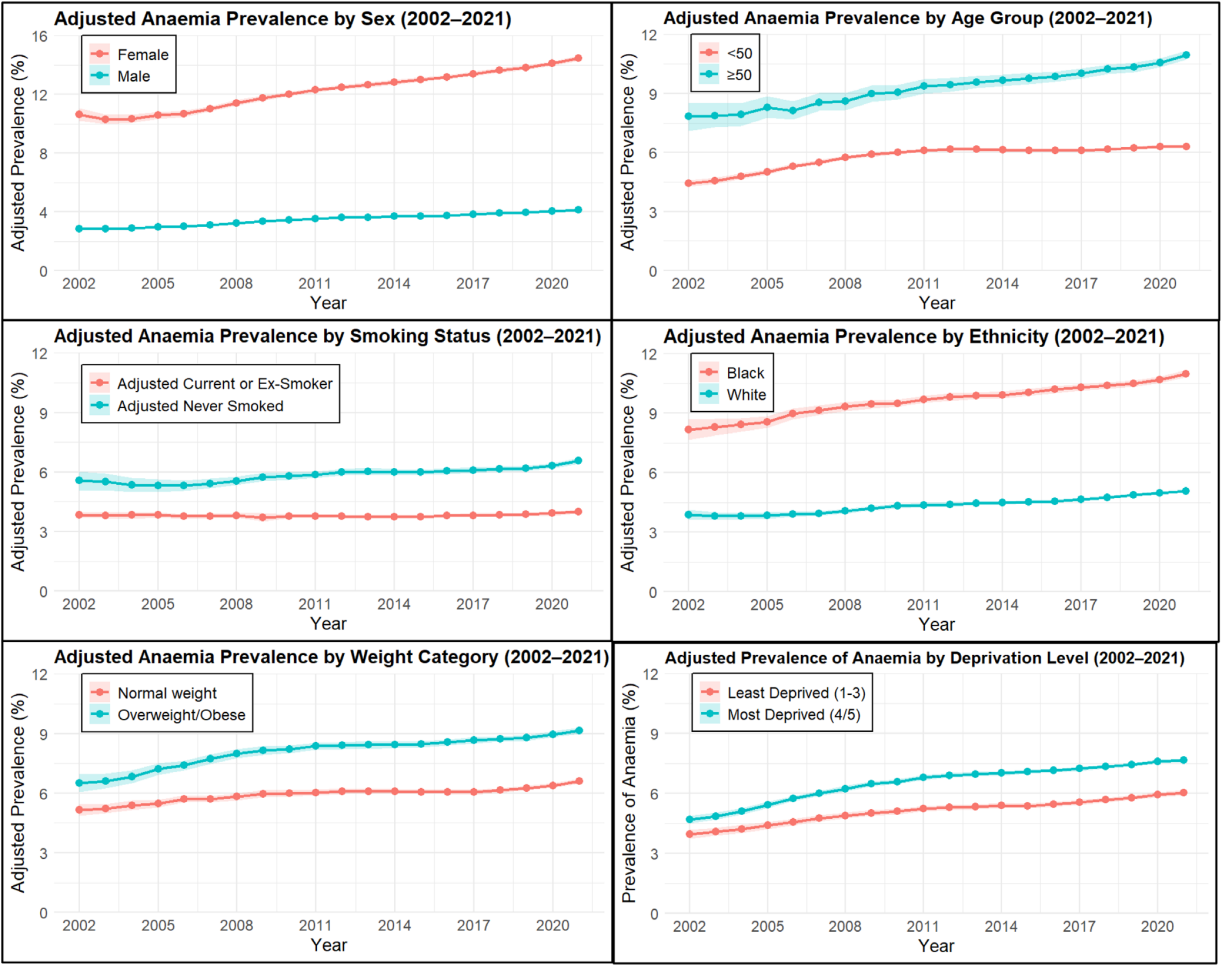
Prevalence of anaemia (y‐axis) in PLWH between 2002 and 2021, with 95% confidence intervals across sub‐groups.

The adjusted prevalence of anaemia among people living with HIV increased from 4.89% (95% CI: 4.72%, 5.06%) in 2002 to 7.42% (95% CI: 7.33%, 7.51%) in 2021 (Figure 2; Data S1). A positive trend was found over time, with the prevalence of anaemia increasing by 0.12% each year on average (Regression coefficient + 0.123; 95% CI: 0.107, 0.139; *p* < 0.001). All sub‐groups of people living with HIV demonstrated significant increases in the prevalence of anaemia over time (Figure 3; Data S1); however, the magnitude of increase was reduced when adjusted. Females living with HIV demonstrated the highest increase per year (adjusted coefficient + 0.224; 95% CI: 0.209, 0.239; *p* < 0.001) and was significantly higher among older (≥50 years) females compared with younger (<50 years) females (Data S1). People living with HIV who were aged ≥50 years, overweight/obese, most deprived and had never smoked, demonstrated a significantly greater prevalence of anaemia within their respective sub‐group. The number of underweight people living with HIV and people living with HIV within the other ethnic group was small, resulting in wide CIs, and considered too uncertain to present; however, annual prevalence and the 95% CIs for these groups and all other sub‐groups can be found in the Data S1.

## DISCUSSION

We present the prevalence and trends of anaemia among people living with HIV from 2002 to 2021. Results indicate an increasing prevalence of anaemia among people living with HIV in the UK overall and among all investigated sub‐groups of people living with HIV. People living with HIV aged ≥50, females, non‐smokers, of Black ethnicity, overweight/obese, and from more deprived backgrounds demonstrated an increased prevalence of anaemia overall and for each study year investigated.

We report an overall prevalence of anaemia of 6.9% among people living with HIV. This differs from the 47% reported by the 2022 meta‐analysis [30]. However, as previously established, the validity of this meta‐analysis is questionable given the considerable heterogeneity (*I* ^2^ = 99.5%) of included studies. Further, many of the included studies were largely not comparable with ours in terms of setting (33 out of 47 were conducted in low‐resourced settings), population (e.g., high‐risk sub‐groups) and measuring and defining anaemia [30]. Of the studies conducted in high‐income countries [30], only one was a population‐based study conducted in France in 2009, and the authors reported a higher prevalence compared with ours (34.5%) [49]. However, this study utilized a threshold of anaemia of <140 g/L in men compared with the <130 g/L recommended by WHO and utilized by GPs in defining anaemia from blood tests in the UK, and therefore, may have overestimated the prevalence of anaemia compared with our study. This study also utilized active screening to measure for anaemia, whereas, ours is based on clinical diagnoses of anaemia recorded in primary care electronic health records, which could explain these discrepancies. A study assessing the prevalence of chronic kidney (CKD) disease using CPRD data has shown that using blood test results to define cases gives double the prevalence as using clinical diagnosis codes, but the greater the severity of CKD, the greater the case detection using diagnosis codes [50]. Therefore, our use of clinical codes to detect cases will likely underestimate the true prevalence of anaemia in people living with HIV, but is likely to more accurately reflect clinically significant anaemia, and is especially valuable for understanding trends in anaemia over time. This highlights an important point that anaemia among people living with HIV may be underdiagnosed in primary care data, which warrants further investigation.

This study is the first to assess trends in the prevalence of anaemia in people living with HIV in the UK, and to our knowledge the first in Europe. Our results differ from a study in North America and a study conducted in China which demonstrated a decreasing trend of anaemia among people living with HIV [36]. This contradiction can be attributed to differences between the sample demographics; the China study comprised mostly farmers from rural areas in a single province in China who were diagnosed with HIV following contaminated blood transfusions [36] and the North America study comprised mostly males (88%). Our sample was population‐based, comprising both rural and urban inhabitants of diverse ethnicities (including 29% from Black ethnic groups which was found to be associated with a higher prevalence), with a larger number of females (32% which was found to be associated with a higher prevalence), and in the UK where HIV is mostly transmitted through sexual intercourse and among key populations [51].

The increasing trend in anaemia we report can in part be attributed to the ageing population of people living with HIV. Between 2002 and 2021, the proportion of people living with HIV aged ≥50 years nearly doubled, and our sub‐group analysis demonstrated that people living with HIV aged ≥50 years experienced a higher prevalence of anaemia than those aged <50 years. Older age in the general population is associated with higher burdens of anaemia which can be classified as nutritional deficiency, anaemia of chronic disease, or unexplained anaemia; the latter is often due to undiagnosed chronic disease. For people living with HIV, an increase in age‐related comorbidities for which many are associated with anaemia, such as cardiovascular disease and chronic kidney disease, may be a key driver behind the increasing trend we report. Additionally, longer duration of living with HIV, and therefore longer time with persistent immune activation and inflammation, and extended exposure to ART (including past exposure to more toxic ART and ART associated with anaemia) are likely contributing factors. Furthermore, even when viral loads are suppressed, similar dysregulation of inflammatory cytokines as seen in the normal ageing process has been found among people living with HIV [52, 53, 54], indicating that people living with HIV may experience premature and accelerated physiological ageing [25, 55]; this may have an impact on anaemia among younger people living with HIV. Further research is needed to identify which of these complex underlying mechanisms contribute to anaemia among people living with HIV to develop effective prevention and treatment plans for people living with HIV, particularly as this vulnerable population lives longer with HIV, is exposed longer to ART, and suffers more from comorbid chronic conditions.

The higher prevalence of anaemia among females living with HIV compared with males is in line with what is known within the general population, that females experience higher rates of anaemia driven by factors such as menstruation and pregnancy [56]. The same may be true regarding the higher prevalence of anaemia in people living with HIV from Black ethnic groups in our study, whereby [57, 58] higher rates of haemoglobinopathies (e.g., sickle cell) within Black ethnic groups are associated with higher rates of anaemia in the general population [59]. Higher BMI is a key risk factor for anaemia in the general population and is indicated by a higher prevalence of anaemia in our sample of people living with HIV [13, 18]. Evidence indicates that smoking can lead to increased haemoglobin levels, likely in response to carbon monoxide [60], which may explain the lower rates of anaemia in this group within our study. Whether HIV exacerbates the prevalence among these sub‐groups cannot be determined without a matched unexposed group of people without HIV. We also report an increasing prevalence of anaemia among these sub‐groups, their counterparts and among people living with HIV overall; thus, further investigations are needed to understand whether HIV‐related mechanisms such as ART use (type of ART or duration of use) or viral load presence (even at low levels) are driving this trend.

The main strength of this study was the use of a population‐based database which produced one of the largest databases of people living with HIV, and is nationally representative. This dataset also enabled us to assess trends over 20 years and among key sub‐groups. However, there are some limitations to mention. Some people living with HIV may opt out of having the sexual health clinic share their HIV diagnosis with their GP, and although HIV prevalence in primary healthcare data is similar to national statistics [45], there could be a slight underrepresentation of people living with HIV in the CPRD dataset used in our study. Full blood count is regularly measured in sexual health clinics for people living with HIV in the UK rather than in primary care settings; therefore, it is unclear whether our results represent an increased burden of mild or severe anaemia, and how types of anaemia have changed over time which is a vital next step to inform resource allocation for optimal service delivery. Similarly, data pertaining to CD4 count, viral load and ART are not well captured in primary care; thus, we were unable to assess trends among people living with HIV with low/high CD4‐count, suppressed/non‐suppressed viral load and exposed to certain ART. Without such data, we are unable to report on how improved CD4 count and viral load or certain ART regimens may have impacted trends of anaemia over time. Data on diet and nutritional status are also not well recorded in primary care records which could potentially be confounders. Lastly, some of the sub‐groups (e.g., underweight people living with HIV and those from ‘other’ ethnic groups) suffered from reduced power, particularly in the earlier years of the study period and, therefore, meaningful interpretation of these findings are not possible.

We report an overall prevalence of anaemia of 6.9% among people living with HIV in the UK, which has increased from 3.9% in 2002 to 8.0% in 2021. This increasing trend was consistent across all sub‐groups of people living with HIV. Female sex, aged ≥50 years, non‐smokers, Black ethnic groups, more deprived backgrounds, and overweight/obese BMI had the highest prevalence of anaemia overall. The ageing population of people living with HIV, in combination with HIV‐related mechanisms, are possible key drivers behind these trends and warrant further investigation. Further research is needed to ascertain clinical and cost‐effective ways to aid in prevention, early diagnosis and targeted treatment of anaemia among people living with HIV. As the average age of people living with HIV continues to increase, these efforts will become increasingly urgent to reduce further morbidity, premature mortality and excess costs on the healthcare system and people living with HIV.

## AUTHOR CONTRIBUTIONS

All authors contributed to the conceptualization of the project. TEG drafted the protocol for CPRD approval and extracted the data from CPRD. JC worked with the internal clinical coding group to develop and review the clinical codes used within the project. JC also provided clinical expertise and guidance on the analysis and interpretation of results. GBF conducted the data analysis and wrote all versions of the manuscript. KN facilitated data access and availability of the clinical coding group. TEG and GNT supervised the project. All authors have reviewed and contributed to the final manuscript.

## CONFLICT OF INTEREST STATEMENT

There are no conflicts of interest to declare.

## ETHICS STATEMENT

All required ethical clearance was obtained. CPRD data undergoes annual ethics approval from the UK Health Research Authority Ethics Committee to receive and supply patient data for public health research. Ethical approval for this data was obtained by the CPRD Independent Scientific Advisory Committee (reference 22_001827).

## Supporting information


**Data S1.** Supporting Information.

## Data Availability

Access to anonymized patient data from CPRD Aurum is subject to a data sharing agreement containing detailed terms and conditions of use following protocol approval from the Independent Scientific Advisory Committee. This study‐specific dataset is therefore not publicly available but can be requested from the corresponding author and subject to research data governance approvals. Details about Independent Scientific Advisory Committee applications and data costs are available on the CPRD website (cprd.com).
